# Adhesive Capsulitis of the Shoulder: Evaluation With US-Arthrography Using a Sonographic Contrast Agent

**DOI:** 10.1038/s41598-017-05491-x

**Published:** 2017-07-17

**Authors:** Xueqing Cheng, Zhenqi Zhang, Guo Xuanyan, Tingting Li, Juan Li, Longlin Yin, Man Lu

**Affiliations:** 1 Department of Ultrasound, Sichuan Cancer Hospital Institute, Sichuan Cancer Center, School of Medicine, University of Electronic Science and Technology of China, Chengdu, China; 2 Department of Ultrasound, Sichuan Academy of Medical Sciences & Sichuan Provincal People’s Hospital, School of Medicine, University of Electronic Science and Technology of China, Chengdu, China; 3 School of Medicine, University of Electronic Science and Technology of China, Chengdu, China; 4 Department of Radiology, Sichuan Academy of Medical Sciences & Sichuan Provincial People’s Hospital, School of Medicine, University of Electronic Science and Technology of China, Chengdu, China

## Abstract

Adhesive capsulitis (AC) is a painful and disabling disorder, which caused restricted motion and chronic pain of shoulder. Intracavitary contrast-enhanced ultrasound has been recently applied to assess obstructive bile duct diseases, tubal patency, vesicoureteric reflux and so on. The aim of this study was to detect the value of US-arthrography by injecting the contrast agent SonoVue into glenohumeral joint compared with US in diagnosing AC. Utrasound (US) and US-arthrography images of 45 patients with AC were compared with that of 45 control subjects without AC with MRI as a gold standard. Patients with AC had a significantly thickened coracohumeral ligment (CHL, 3.1 mm) and inferior capsule (3.5 mm) on US, and a decreased volume of axillary recess (1.14 ml) on US-arthrography compared with the control subjects (1.59 ml). Filling defect (91.1%) and synovitis-like abnormality (75.6%) in the joint on US-arthrography were more sensitive than that of rotator interval abnormality (71.1%), thickened CHL more than 3 mm (64.4%), thickened inferior capsule more than 3.5 mm (66.7%) on US respectively for diagnosis of AC. Consequently, US-arthrography was more effective method than US for assessment of AC. Filling defects of joint cavity and synovitis-like abnormality in the joint are characteristic US-arthrography findings for diagnosing AC.

## Introduction

Adhesive capsulitis of the shoulder (AC), also known as frozen shoulder, is an inflammatory and fibrosing condition of the shoulder characterised by progressive pain and decreased range of motion of the glenohumeral joint^1, 2^. It is a said to be a self-limiting condition lasting for an average of 2–3 years, but up to 40% of patients may have persistent symptoms and restricted movement beyond 3 years, with 15% left with permanent disability^3, 4^. The etiology is still unknown, which may be associated with diabetes mellitus, Dupuytren disease, shoulder trauma, various cardiac, endocrine, and neurologic disorders^5, 6^. Although AC is much less common than subacromial impingement syndrome, calcific tendinitis and rotator cuff tear, it is difficult to differentiate them from each other based on clinical presentation sometimes. So it is of great importance to explore some characteristic imaging findings of AC in order to make an accurate diagnosis and choose the treatment further.

Arthrography, ultrasound (US), plain magnetic resonance imaging (MRI) and MR arthrography (MRA) may provide reliable imaging indicators of frozen shoulder^7^. The traditional shoulder arthrography was used to assess the volume of the glenohumeral joint. Both US and MRI have high soft-tissue resolution and are widely used to assess coracohumeral ligament (CHL) and capsule and synovium for AC^8–10^. Numerous specific MRI/MRA findings have been reported to diagnose AC, which includes thickening of the CHL, thickening of the joint capsule in the rotator cuff interval and axillary recess, effacement of the axillary recess, obliteration of subcoracoid fat triangle and so on^7, 10–13^.

Recently, contrast enhanced ultrasound (CEUS) has been widely applied in clinical disease diagnosis by using microbubbles intravenously or intracavitaryly, especially the intravenous use to image real time tissue perfusion and characterize the vasculature of lesions or an organ of interest. Some intracavitary use of microbubbles have also been reported to reflect other lesions such as obstructive bile duct diseases, tubal patency, vesicoureteric reflux and so on^14, 15^. Cheng *et al*.^16^ described a new application of CEUS called percutaneous ultrasound-guided subacromial bursography (PUSB) to assess rotator cuff tears by injecting microbubbles into the subacromial bursa.

In this study, we firstly applied US-arthrography by performing CEUS by injecting the contrast agent SonoVue into glenohumeral joint to assess frozen shoulder. The purpose of our study, therefore, was to evaluate the effect of US-arthrography compared with traditional ultrasound in diagnosing adhesive capsulitis.

## Methods

### Study Population

Our hospital’s ethics committee approved this study (SCCHIEC-D-2013 (087)). Informed consent was obtained from the patients at the time of examination about the possibility that their medical records and images will be reviewed and published for scientific purposes. Between October 2013 and September 2015, a total of 96 patients with frozen shoulder were performed with US and US-arthrography at our hospital. Forty-five patients were included in our retrospective analysis on the basis of the following criteria: (a) clinical diagnosis of frozen shoulder, and MRI confirmation of the diagnosis; (b) MR examination performed less than 24 hours after US-arthrography; (c) US and US-arthrography of the shoulder performed at our institution according to a standardized protocol. Exclusion criteria were: (a) precious shoulder surgery or systemic inflammatory arthritis (e.g., rheumatoid arthritis, seronegative spondyloarthropathy, and psoriatic arthritis); and (b) an additional diagnosis of rotator cuff tear. Of the 51 excluded patients, 33 didn’t undergo MRI within 24 hours after US-arthrography, 10 had an additional rotator cuff tear with frozen shoulder, 5 didn’t indicate any features of frozen shoulder by MRI, 2 had rheumatoid arthritis, and 1 had a history of shoulder trauma.

There were 30 females and 15 males, with ages ranging from 46 to 63 years (mean age 54 years). The right shoulder was affected in 25 of the 45 (25/45) cases, and the left in 20 (20/45) patients. The mean duration of symptoms was 10 months (range, 1.5–24 months). For patient that MR imaging indicated any of these three features: (a) thickness of CHL ≥ 4 mm, (b) thickness of capsule and synovium of the axillary recess greater than 3 mm, and (c) subcoracoid triangle sign was confirmed as frozen shoulder by MRI in this study. Twenty-six patients meet one item, 12 patients meet two, and 7 meet three of the criteria respectively.

The control group consisted of 45 patients who underwent US-arthrography no more than 24 hours prior to MRI but who didn’t show any clinical or MRI feature of frozen shoulder. The indication for MRI were rotator cuff tears (n = 16), shoulder impingement syndrome (n = 20), biceps tendon lesion (n = 6), and superior labrum anterior-posterior lesion (n = 3). There were 24 men and 21 women, aging from 50 to 68 years (mean age 62 years).

### US Protocol

All US examinations were performed by one radiologist (G.X.Y, with more than 5 years of experience in musculoskeletal US) using a 5–12 MHz transducer (Philips iU-Elite ultrasound system, Bothell, Wash). In this study, the CHL, rotator interval, and inferior glenohumeral (GH) capsule were scanned in sequence with patients sit in a chair. For assessment of CHL, the transducer was placed on the lateral border of the coracoid process to obtain a longitudinal image and to measure the thickness of the CHL (the thickest portion) with the shoulder in a neutral position and the forearm extended^8^. The rotator interval was scrutinised with the patient’s fist held by the side to detect if there were any increased echotexture or increased vascularity by using grey-scale and color Doppler sonography^17^. While the thickest portion of inferior capsule was measured on the axial plane with the shoulder in maximal abduction and neutral rotation^7^.

### US-arthrography Protocol

All US-arthrography examinations were performed by one radiologist (L.M, with more than 5 years of experience in musculoskeletal ultrasound and CEUS) using a 3–9 MHz linear array transducer (Philips IU-Elite ultrasound system, Bothell, Wash) designed for contrast harmonic imaging.

**Figure 1:**
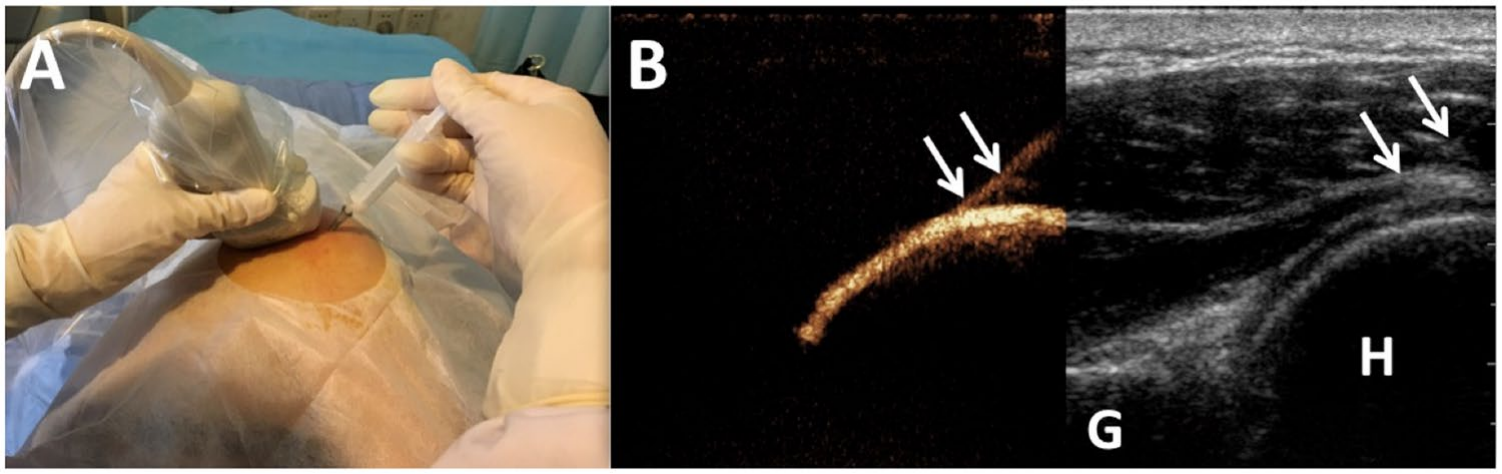
US-guided injection of GH joint for performing US-arthrography. **A**, Patient positioned lateral recumbent position with affected side up; **B**, US-arthrography showed that the contrast media was accurately injected into GH joint, arrows showed the needle. G, Glenoid fossa; H, Humeral.

A standard protocol of US-arthrography was performed as follows. Initially, participants lay on the examination table in a lateral recumbent position with the affected shoulder side up (Fig. 1A). First, 5 ml sodium chloride solution (9 mg/ml) was injected into a bottle of SonoVue (Bracco, Milan, Italy) to make dispersion. After aseptic preparation, the contrast harmonic imaging mode was activated, and then the GH joint was injected with a 22-gauge needle (BD, Shanghai, China) by using an in-plane posterolateral ultrasound-guided injection technique (Fig. 1B). The solution injected was consisted of 1 mL of SonoVue dispersion, 1 ml lidocaine (20 mg/ml), and 18 ml sodium chloride (9 mg/ml). A maximum volume of 20 ml fluid was totally injected until the plunger became difficult to depress or the patient complained of pain. The MI (mechanical index) value displayed on the screen was less than 0.2 which exploits the harmonic properties of the microbubbles without rupturing them, and a pulse inversion technique. The volume of intraarticular injection was recorded. After the injection, patients were asked to seat at the edge of examination table. The distribution of mixture in the GH joint and the capsule were dynamically observed from posterolateral, anterior and inferior aspect of the shoulder in neutral position. The rotator cuff and subacromial-subdeltoid bursa was routinely scanned to detect if there presented any contrast mixture. Images and videos were stored on the hard drive of the system for documentation and analysis. All patients were observed for 30 minutes to rule out adverse events, and rechecked by contrast harmonic imaging mode to confirm if there were any visible hyperechoic microbubbles at the end of US-arthrography.

### MRI Protocol

MRI was performed with a 1.5 T MR system (Signa Advantage; General Electric Medical Systems, Milwaukee, WI or Magnetom Vision, Siemens Medical System, Erlangen, Germany) with appropriate surface coils for shoulder imaging. Patients were positioned with the humeral in a neutral position. MRI protocols included the following: oblique coronal fast T2-weigthed images with fat saturation (TR/TE: 3000 54 ms) and T1-weighted images (TR/TE: 500/minimalms), oblique sagittal fast T2-weighted images (TR/TE: 3000/90 ms), axial gradient echo images (TR/TE: 450/20, 20 flip angle), and fast T2-weighted images with fat saturation (TR/TE: 3000/50 ms). All the sequences used a matrix of 256 × 192, two excitations, a 14 cm field of view (FOV), a 4 mm section thickness and 0.4 mm intersection gap. For the fast T2-weighted images, the echo trains were 8.

### Images Analysis

US and US-arthrography images were analyzed in consensus by two radiologists (G.X.Y and L.M), who were blinded to patients’ demographic date, history, group, and MR findings. For US evaluation, thickness of CHL and inferior glenohumeral capsule were obtained, rotator interval abnormality and biceps sheath effusion was observed and characterized as present or absent^7, 8, 18, 19^. Rotator interval abnormality was diagnosed with the detecting of hypoechoic echotexture or increased vascular flow in rotator interval.

**Figure 2:**
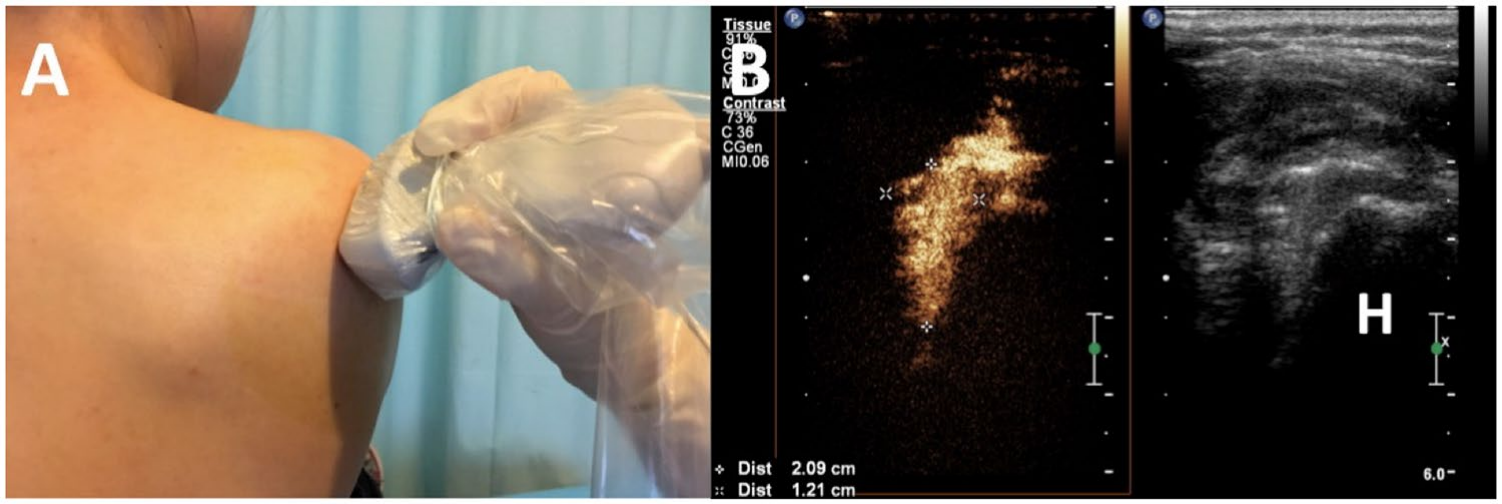
Transducer position and measurement at the long-axis view of the axillary recess on US-arthrography. **A**, Transducer was placed at the anatomic sagittal oblique plane with patient seated at the edge of examination table; (**B**). Calipers were set to measure the maximal height (+) and depth (×) of the axillary recess. H, Humeral.

**Figure 3:**
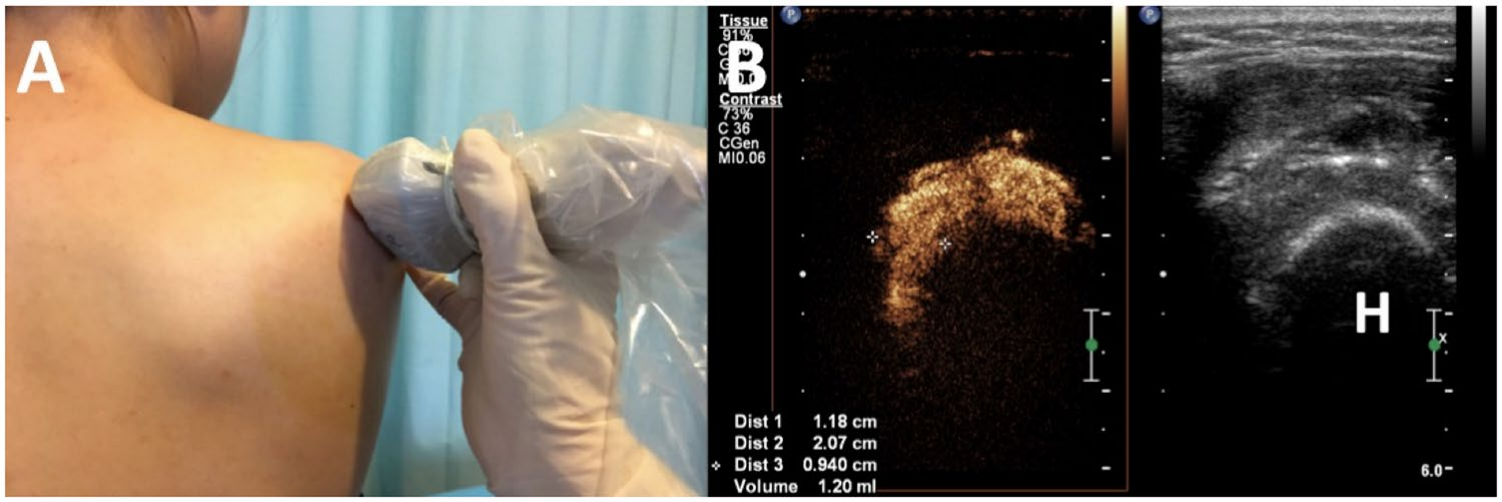
Transducer position and measurement at the short-axis view of the axillary recess on US-arthrography. **A**, Transducer was placed at the anatomic axial oblique plane with patient seated at the edge of examination table; (**B**). Calipers were set to measure the maximal width (+) of the axillary recess. The volume was automatically calculated by using the equation of v = 0.52 hwd on the US machine. H, Humeral.

**Figure 4:**
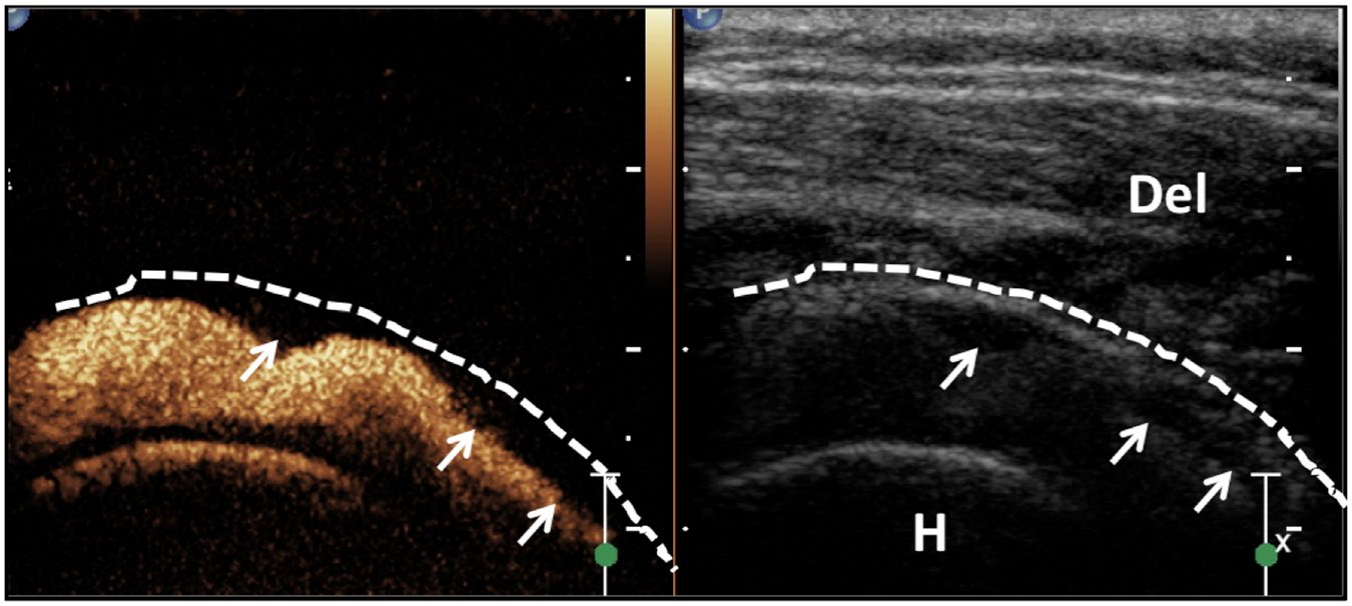
US-arthrography of a 62-year-old woman with frozen shoulder for about 6 months demonstrated the finding of intraarticular filling defects (arrowheads). The dotted line outlined the contour of capsule. Del, Deltoid.

**Figure 5:**
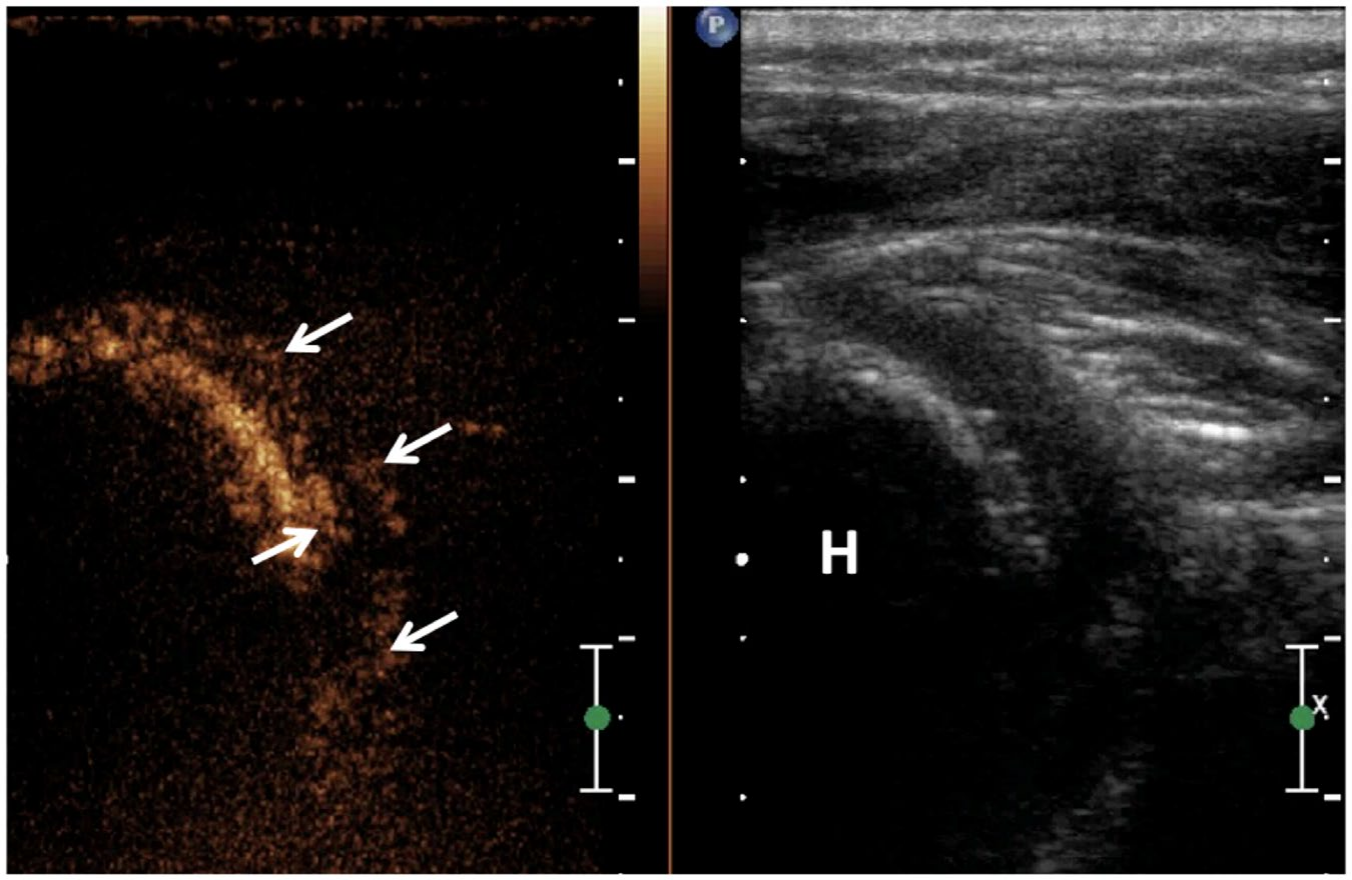
US-arthrography of a 60-year-old woman with frozen shoulder for about 3 months demonstrated the finding of synovitis-like abnormality in the joint (arrowheads). Hyperechoic microbubbles were retained on the synovium debris floating in the joint fluid (arrowheads).

Both quantitative and qualitative criteria were used for analysis of US-arthrography. Quantitative criteria included volume of the injection fluid, the maximal height and depth of the axillary recess were determined on long-axis view (anatomic sagittal oblique plane) images (Fig. 2), and the width of this structure was determined on transverse (anatomic axial oblique plane) images (Fig. 3). The volume of the axillary recess was calculated in milliliters by using the equation for elliptical volume, v = 0.52 (hwd), where h is height, w is width, and d is depth. The following qualitative criteria were evaluated and characterized as present or absent: (a) filling defects of joint cavity (Fig. 4), (b) synovitis-like abnormality in the joint, (c) extravasation of contrast material into the muscle around the needle track, (d) extravasation of contrast material into rotator cuff and/or subacromil-subdeltoid (SASD) bursa. Synovitis-like abnormality was diagnosed on the basis of evidence of synovial irregularity and/or fibrous debris floating in the joint fluid (Fig. 5).

The MR images were analyzed by one single musculoskeletal radiologist (Y.L.L) with 10 years experiences. Any of the following MR findings indicated a presence of adhesive capsulitis in this study: (a) thickening of the CHL to 4 mm or more, (b) thickening of the capsule and synovium greater than 3 mm in the axillary recess, (c) partial or complete obliteration of the subcoracoid fat triangle^9, 13^.

### Statistical Analysis

Statistical analysis of between-group differences in quantitative data which included thickness of the CHL, the inferior glenohumeral capsule, volume of the intraarticular injection, volume of the axillary recess (including height, width and depth) was performed using the Mann-Whitney test or the Kruskal-Wallis test (α = 0.05, two-tailed). The threshold values of thickened CHL and inferior capsule was obtained by Receiver operating characteristic (ROC) curve. For all other qualitative data comparisons, χ^2^ test was used. Sensitivity and specificity, with 95% confidence intervals, were calculated for those US and US-arthrography criteria and for different cutoff values of measurement of the thickness of the CHL and the inferior capsule on US.

A *P* value of less than 0.05 was considered to indicate a statistically significant difference.

## Results

### US assessment

**Figure 6:**
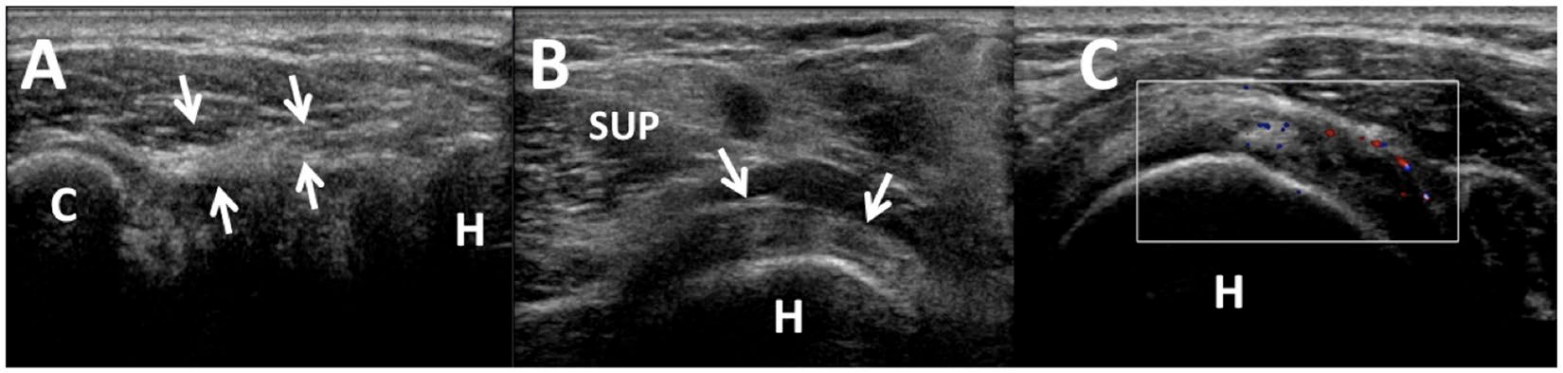
US findings of frozen shoulder patients: (**A**) Gray-scale US showed a thickened CHL (arrowheads); (**B**) Gray-scale US showed a thickened inferior capsule; (**C**) Color Doppler detected an increased vascularity and echotexture in the rotatol cuff interval; C, coracoid; SUP, suparaspinatus tendon; H, humeral head.

US showed a significant mean thickened CHL of AC patients than that of the control subjects (3.1 ± 0.67 mm vs 1.4 ± 0.55 mm, *P* < 0.01) (Fig. 6A, Table 1). A threshold value of 3 mm or more had a specificity of 88.9% and a sensitivity of 64.4% for the diagnosis of frozen shoulder. In patients with frozen shoulder, the inferior capsule was significantly thickened (3.5 ± 1.06 mm vs 1.6 ± 0.72 mm, *P* < 0.01) (Fig. 6B). A threshold value of 3.5 mm or more had a specificity of 93.3% and a sensitivity of 66.7% for the diagnosis of frozen shoulder. There were 32 patients demonstrated abnormality in rotator interval (Fig. 6C). However only 3 out 45 patients in the control group demonstrated either hypoechoic echotexture or vascularity within the rotator interval (Table 2). US detected abnormality in the rotator interval had a sensitivity of 71.1% and a specificity of 92.5% for diagnosis of AC. Biceps tendon sheath effusion was not significantly more frequently in patients than in control subjects (55.6% vs 48.9%, *P* = 0.53) (Table 2).

**Table 1:** Quantitative Criteria of US and US-arthrography for Assessing Frozen Shoulder.

Criterion	Patients with Frozen Shoulder (n = 45)	Control Group (n = 45)	*P* Value
**US**			
Thickness of CHL	3.1 ± 0.67	1.4 ± 0.55	<0.001
Thickness of inferior capsule	3.5 ± 1.06	1.6 ± 0.72	<0.001
**USA**			
Volume of injection (ml)	18.3 ± 0.29	19.0 ± 0.22	0.07
Axillary recess			
Volume	1.14 ± 0.13	1.59 ± 0.08	0.006
Height	2.0 ± 0.05	2.44 ± 0.05	<0.001
Width	0.98 ± 0.03	1.17 ± 0.02	<0.001
Depth	1.02 ± 0.04	1.05 ± 0.03	0.50

**Table 2:** Qualitative Criteria of US and US-arthrography for Assessing Frozen Shoulder.

Criterion	Patients with Frozen Shoulder (n = 45)	Control Group (n = 45)	*P* Value
**US**			
Rotator interval abnormality	32	3	<0.001
Biceps tendon sheath effusion	25	22	0.53
**USA**			
Filling defect of joint cavity	41	6	<0.001
synovitis-like abnormality in the joint	34	10	<0.001
CA leakage around the needle	13	8	0.21
CA leakage into rotator cuff and/or SASD bursa	0	14	<0.001

### US-arthrography assessment

US-arthrography was successfully performed among all the participants. Contrast media extravasations occurred more frequently in AC patients than in control group (44.4% vs 22.5%, *P* = 0.01) (Table 2), but did not negatively affect the diagnostic quality of the US-arthrography image. In the AC group, a mean volume of 18.3 ml (range from 12 ml to 20 ml) contrast mixture was injected until the manipulator felt a great resistance. There were of no significant differences between the control group and the patient group in injection fluid volume (19.0 ± 0.22 ml vs 18.3 ± 0.29 ml, *P* = 0.07). The volume of the axillary recess, however, was significantly smaller in patients with frozen shoulder than in control subjects (1.14 ± 0.13 ml vs 1.59 ± 0.08 ml, *P* < 0.01), because of the significantly smaller height and width of the axillary recess in patients (Table 1).

Filling defects of joint cavity and synovitis-like abnormality in the joint were seen significantly more frequent in patients than in control subjects (91.1% vs 13.3% and 75.6% vs 22.2%, respectively, both *P* < 0.05) (Table 2). Filling defects of joint cavity showed a greater sensitivity and a greater specificity (86.7%) than synovitis-like abnormality in the joint for the diagnosis of frozen shoulder (91.1% vs 75.6%, 86.7% vs 77.8%, *P* < 0.05) (Table 3). The leakage of contrast mixture from glenohumeral joint into rotator cuff and/or overlying subacromial-subdeltoid bursa was observed in 14 control subjects with rotator cuff tear, but not observed in anyone in AC group (Table 2).

**Table 3:** Diagnostic Value of Significant Quantitative and Qualitative Criteria for Diagnosis of Frozen Shoulder.

Criterion		Sensitivity		Specificity	
		Percentage(%)	95% CI (%)	Percentage	95% CI (%)
**Quantitative**					
US	≥3 mm thickness of CHL	64.4	(48.8, 78.1)	88.9	(76.0, 96.3)
	≥3.5 mm thickness of inferior capsule	66.7	(51.1, 80.0)	93.3	(81.7, 98.6)
**Qualitative**					
US	Rotator interval abnormality	71.1	(55.7, 83.6)	92.5	(81.7, 98.6)
USA	Filling defect of joint cavity	91.1	(78.8, 97.5)	86.7	(73.2, 95.0)
	Synovitis-like abnormality in the joint	75.6	(60.5, 87.1)	77.8	(62.9, 88.8)

## Discussion

Synovial inflammation and capsular fibrosis are the central pathology of adhesive capsulitis (AC), which subsequently leads to formation of adhesions, capsular contracture and decreased joint capacity^6^. In the past decades, several studies had been performed to assess the usefulness of MRI and MRA in the diagnosis of frozen shoulder^9, 11, 13, 18, 20^. MRI and MRA are both capable of diagnosing adhesive capsulitis as well as determining the pathophysiological stage and ruling out concurrent pathology^18^. However, MRI needs a relative long time for examination and reservation and was unavailable in patients with non-MR-compatible hardware. MRA is invasive, it requires an additional procedure for intraarticular injection of contrast media under guidance of US or fluoroscopy.

While, ultrasound is especially advantageous because it allows lower cost, faster examination, dynamic assessment and ease of access. Homsi *et al*.^8^ concluded that US was access to determine CHL thickness and the average thickness is significantly greater in adhesive capsulitis shoulders (3 mm) than in painful shoulders (1.39 mm) and asymptomatic shoulders (1.34 mm). Michelin *et al*.^7^ also used US to measure the inferior glenohumeral capsule thickness with a transducer placed within the axilla in maximally abducted shoulders. They found that the inferior capsule was thickened in shoulders with capsular contracture. US can be used to evaluate the hypervascular synovium and fibrovascular scar tissue in the rotator interval for frozen shoulder. Enhanced vascularity and hypoechoic change within the rotator interval were thought as useful criteria for the sonographic diagnosis of adhesive capsulitis^17^. In our study, the mean thickness of both CHL and inferior capsule in AC patients were significantly greater than that in non-AC patients. A thickened CHL more than 3 mm on US indicated a low sensitivity of 64.9%, but high specificity of 93.3%. While a thickened inferior capsule more than 3.5 mm on US indicated a sensitivity of 66.7% and a specificity of 92.5%. Abnormality in the rotator interval on US demonstrated a sensitivity of 71.1% lower than the reported 97% by Lee *et al*.^18^, it may because that the patients included in the previous study all had a history less than one year and were correlated with arthroscopy.

However, CHL was sometime invisible for reasons like anatomic variant and restricted scanning position^8, 19^, while a thickened inferior capsule may be difficult to discriminate on US without joint distension when patients had a severe limitation of abduction. Conventional ultrasound was more experience-dependent.

For performing US-arthrography, we distend the capsule by injecting ultrasonic contrast agent mixture into the glenohumeral joint under real-time ultrasound guidance firstly. Then we could make both quantitative and qualitative analysis of this procedure in a way similar to MRA. With nearly the same volume of injection fluid of the two groups, AC patients seemed to have a higher incidence of extravasation and a decreased volume of axillary recess than non-AC patients in the control group. This finding of a significantly smaller volume of axillary recess corresponds with results of the previous study using conventional arthrography and MR arthrography^9^. Mengiardi *et al*.^9^ just hypothesized that it may represent a reduced volume of intraarticular contrast material or early leakage of contrast material caused by weakening in the joint capsule. In our study, we documented the exact amount of contrast mixture injected into each patient and demonstrated that the volume of injection was not significantly reduced for AC patients compared with the control subjects. The decreased volume of axillary recess could possiblely attribute to the contracture and adhesions of capsule.

US-arthrography also allowed qualitative analysis of synovitis and capsule adhesion for adhesive capsulitis. The finding of filling defects of joint cavity was mostly detected in AC patients which may related to the irregular thickening with distortion of normal capsule and synovium, with a sensitivity of 91.1% and a specificity of 86.7%. In our study, the observation of hyperechoic microbubbles retaining on the debris in the joint or on the capsule subsynovial layer until most of microbubbles in joint were invisible was regard as synovitis-like abnormality. Lindner^21, 22^ had reported that the lipid and albumin microbubbles could retain within the microcirculation of inflamed tissue because of their attachment to activated leukocytes. Therefor, the finding of synovitis-like abnormality may represent a hypervascular synovitis. There were 10 control subjects detected with a synovitis-like abnormality in the joint, which demonstrated a co-existence of synovitis in the GH joint. Although US-arthrography is invasive as compared to US, US-arthrography finding of filling defects and synovitis-like abnormality in the joint showed greater sensitivity than US findings in this study. Filling defects of joint cavity on US-arthrography was the most sensitive (91.1%) and most specific (86.7%) finding for the diagnosis of adhesive capsulitis.

The main limitation of this study was that we used the clinical and MR finding as the reference standard for the diagnosis of adhesive capsulitis. It excluded those early adhesive capsulitis patients with negative MR finding, which may resulted a selection bias. Second, as asymptomatic volunteers for US-arthrography were not available, the control groups included patients with rotator cuff tear, impingement syndrome, biceps tendon lesion or labral lesion although the controls did not show evidence of adhesive capsulitis on MRI. But to our knowledge, patients with rotator cuff tear especially full-thickness tear may possibly had a secondary frozen shoulder^18^. Lastly, MR examination was performed after distending the GH joint with microbubble contrast agent (actually normal saline because microbubbles was not detected by US-arthrography in several minutes) that showed low signal on T1WI and high signal on T2WI, rather than the gadolinium-containing contrast agents. So the gold standard we used was not the conventional MRI or MRA that most of the previous studies performed.

In conclusion, US-arthrography is a new form of shoulder arthrography by using CEUS and microbubble contrast agents. Intra-articular filling defects and synovitis-like abnormality in the joint are characteristic US-arthrography findings, which are more sensitive than US for diagnosing adhesive capsulitis.
